# Toll-Like Receptors 2 and 4 Regulate the Frequency of IFNγ-Producing CD4^+^ T-Cells during Pulmonary Infection with *Chlamydia pneumoniae*

**DOI:** 10.1371/journal.pone.0026101

**Published:** 2011-11-09

**Authors:** Nina Wantia, Nuria Rodriguez, Christine Cirl, Tanja Ertl, Susanne Dürr, Laura E. Layland, Hermann Wagner, Thomas Miethke

**Affiliations:** Institut für Medizinische Mikrobiologie, Immunologie und Hygiene, Technische Universität München, Munich, Germany; Charité-University Medicine Berlin, Germany

## Abstract

TLR2 and TLR4 are crucial for recognition of *Chlamydia pneumoniae in vivo*, since infected TLR2/4 double-deficient mice are unable to control the infection as evidenced by severe loss of body weight and progressive lethal pneumonia. Unexpectedly, these mice display higher pulmonary levels of the protective cytokine IFNγ than wild type mice. We show here, that antigen-specific CD4^+^ T-cells are responsible for the observed IFNγ-secretion *in vivo* and their frequency is higher in TLR2/4 double-deficient than in wild type mice. The capacity of TLR2/4 double-deficient dendritic cells to re-stimulate CD4^+^ T-cells did not differ from wild type dendritic cells. However, the frequency of CD4^+^CD25^+^Foxp3^+^ T-cells was considerably higher in wild type compared to TLR2/4 double-deficient mice and was inversely related to the number of IFNγ-secreting CD4^+^ effector T-cells. Despite increased IFNγ-levels, at least one IFNγ-mediated response, protective NO-secretion, could not be induced in the absence of TLR2 and 4. In summary, CD4^+^CD25^+^Foxp3^+^ regulatory T-cells fail to expand in the absence of TLR2 and TLR4 during pulmonary infection with *C. pneumoniae*, which in turn enhances the frequency of CD4^+^IFNγ^+^ effector T-cells. Failure of IFNγ to induce NO in TLR2/4 double-deficient cells represents one possible mechanism why TLR2/4 double-deficient mice are unable to control pneumonia caused by *C. pneumoniae* and succumb to the infection.

## Introduction

The obligate intracellular bacterium *Chlamydia pneumoniae* infects the respiratory tract and replicates in bronchial epithelial cells [1], [2]. The cells are infected by elementary bodies of *C. pneumoniae* which develop within hours *post infectionem* into reticulate bodies [1]. The latter form divides several times and within 48 to 72 h a microscopically visible intracellular inclusion is generated. *In vitro*, the replication of *C. pneumoniae* is impaired by IFNγ [3], [4]. This cytokine exerts its effect indirectly via the induction of two enzymes: the inducible isoform of the nitric oxide synthase (iNOS) and indolamine 2,3 dioxygenase (IDO). The former enzyme generates nitric oxide (NO), which is toxic for bacteria and impairs replication of *C. pneumoniae* [5], while the latter degrades the aminoacid tryptophan, which is required by *C. pneumoniae* [6]. *In vivo*, the replication of the bacterium is also controlled by IFNγ, as chlamydial burden in IFNγ receptor-deficient mice is significantly higher than in wild type mice [7]. Based on these findings it is obvious that IFNγ is crucial to control and confine pneumonia caused by *C. pneumoniae*.

The cellular source of IFNγ *in vivo* during pulmonary infection with *C. pneumoniae* was analyzed by Rothfuchs et al [8]. Accordingly, NK cells neither contributed to IFNγ-secretion by bronchoalveolar lavage mononuclear cells nor protected mice. In contrast, IFNγ-secreting CD4^+^ or CD8^+^ T-cells were protective since they impaired replication of *C. pneumoniae*. Thus, IFNγ-secreting cells of the adaptive immune system contribute to host defense against the bacterium.

Innate immune cells like bone marrow derived dendritic (BMDC) cells recognize *C. pneumoniae* via TLR2 and 4 [9]. In contrast to wild type animals, mice double-deficient for TLR2 and 4 were unable to control the replication of the bacteria and succumbed to progressive pneumonia [10]. Moreover, although many immune responses *in vivo*, such as the secretion of pro-inflammatory cytokines and chemokines, depended almost exclusively on TLR2, the survival of infected mice required the presence of TLR2 but also of TLR4 [10]. Unexpectedly, TLR2/4 double-deficient but not TLR2-deficient mice displayed upon infection with the microorganism significantly higher pulmonary levels of IFNγ than wild type mice [10].

TLRs also influence the adaptive immune response. Thus, mice lacking MyD88, the most important adapter molecule of the TLR-signaling cascade, failed to mount a TH1 response upon immunization with the model antigen ovalbumin in complete Freund's adjuvant while antigen-specific TH2 responses were not impaired [11]. In particular, antigen-specific T-cells from MyD88-deficient mice were unable to produce IFNγ, but secreted TH2 cytokines like IL-13 and IL-4 at least as efficiently as T-cells from wild type mice. Likewise, it was shown that endotoxin-stimulated wild type dendritic cells induced allogeneic CD4^+^ T-cells to secrete IFNγ while MyD88-deficient allogenic dendritic cells only stimulated a TH2 response [12]. However, upon vaccination of MyD88-deficient mice with *Mycobacterium bovis* BCG a TH1 response was observed as in wild type mice [13]. Interestingly, the adaptive immune response induced by the vaccination was only partially effective to prevent the lethal outcome of a challenging *Mycobacterium tuberculosis* infection in MyD88-deficient mice. In summary, the influence of MyD88 on adaptive immune responses appears to depend substantially from the model system used.

Here, we explored adaptive immune responses in mice lacking TLR2 and TLR4 upon pulmonary infection with *C. pneumoniae*. The results demonstrate that an increased IFNγ-release by the adaptive immune system in the absence of both TLRs was associated with a lower frequency of regulatory T-cells. However, the cytokine was unable to trigger the release of NO by TLR2/4 double-deficient bone marrow-derived macrophages (BMDM). The latter finding presumably contributes to the increased lethality observed in TLR2/4 double-deficient mice.

## Results

### Pulmonary recruitment of T-cells was not impaired in TLR2/4 double-deficient mice

**Figure 1:**
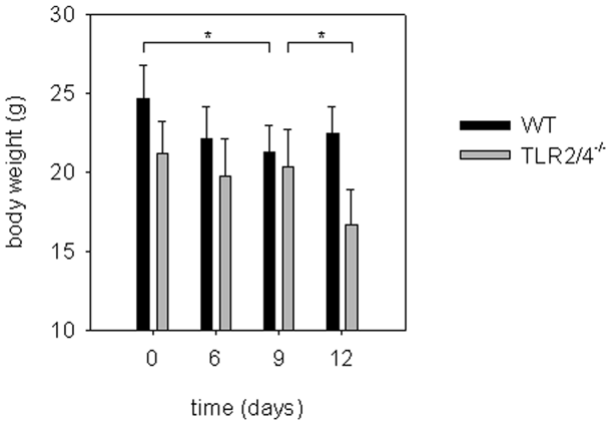
TLR2 and 4 are crucial to control pneumonia-induced loss of body weight of *C. pneumoniae*-infected mice. Wild type (n = 24 d0, n = 18 d6, n = 12 d9, n = 6 d12) and TLR2/4 double-deficient mice (n = 24 d0, n = 18 d6, n = 12 d9, n = 6 d12) were infected with *C. pneumoniae*. At the time point indicated in the graph the body weight of the mice was determined. Bars represent mean and SD of individual mice from two independent experiments. *p<0.001, ANOVA posthoc Holm-Sidak.

**Figure 2:**
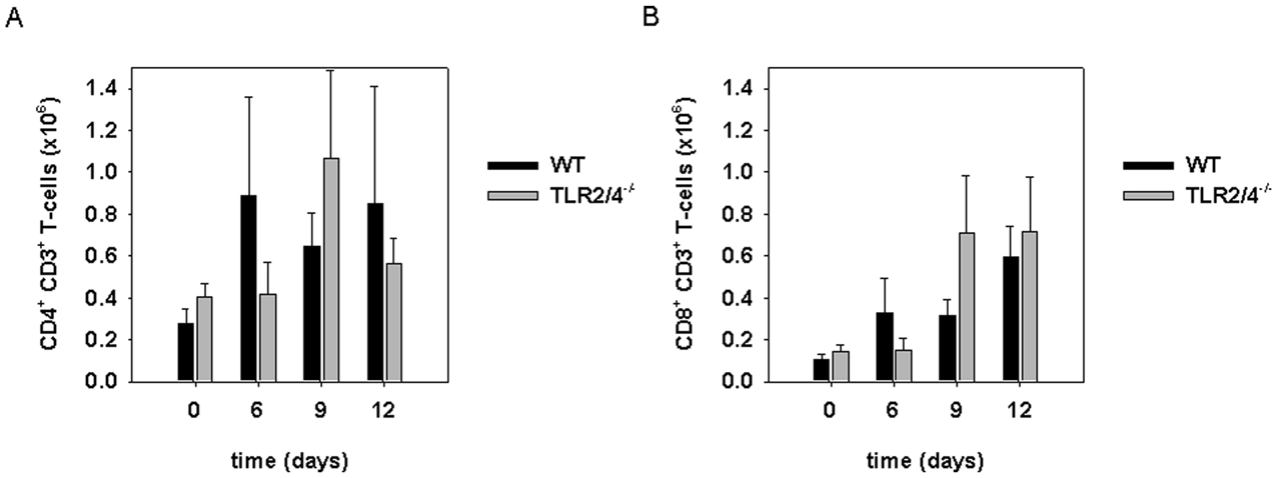
Lack of TLR2/4 does not impair pulmonary recruitment of CD4^+^ and CD8^+^ T-cells upon infection with *C. pneumonia*. Wild type (n = 3/time point) and TLR2/4 double-deficient mice (n = 3/time point) were infected with *C. pneumoniae*. At the time points indicated in the graph mice were sacrificed, lungs removed, single cell suspensions were prepared and the number of cells determined. Cells were stained with mAbs specific for CD4, CD8 and CD3 as described in Materials and Methods, analyzed by flow cytometry and the number of each subpopulation was calculated. CD4^+^CD3^+^ T-cells are depicted in (A), CD8^+^CD3^+^ T-cells in (B). Error bars represent SD of three individual mice.

To explore whether adaptive immune responses were induced *in vivo* in the absence of TLR2 and 4 known to be of key importance for the recognition of *C. pneumoniae*, we infected wild type and TLR2/4 double-deficient mice pulmonary with *C. pneumoniae*. As shown in Fig. 1 these TLR-deficient mice lost a considerable part of their body weight between day 9 and day 12 post infection. In contrast, this was not observed in wild type mice which displayed a weaker weight loss between day 0 and 9. Similar data were demonstrated previously and that study also showed that the TLR2/4 double-deficient mice succumbed to the infection [10]. While recruitment of polymorphonuclear neutrophils into the infected lungs was considerably impaired in TLR2/4 double-deficient mice [10], CD4^+^ and CD8^+^ T-cells were present in wild type and TLR2/4 double-deficient mice in similar numbers (Fig. 2A, B).

### Presence of CD4^+^IFNγ^+^ T-cells in the lung of TLR2/4 double-deficient mice

**Figure 3:**
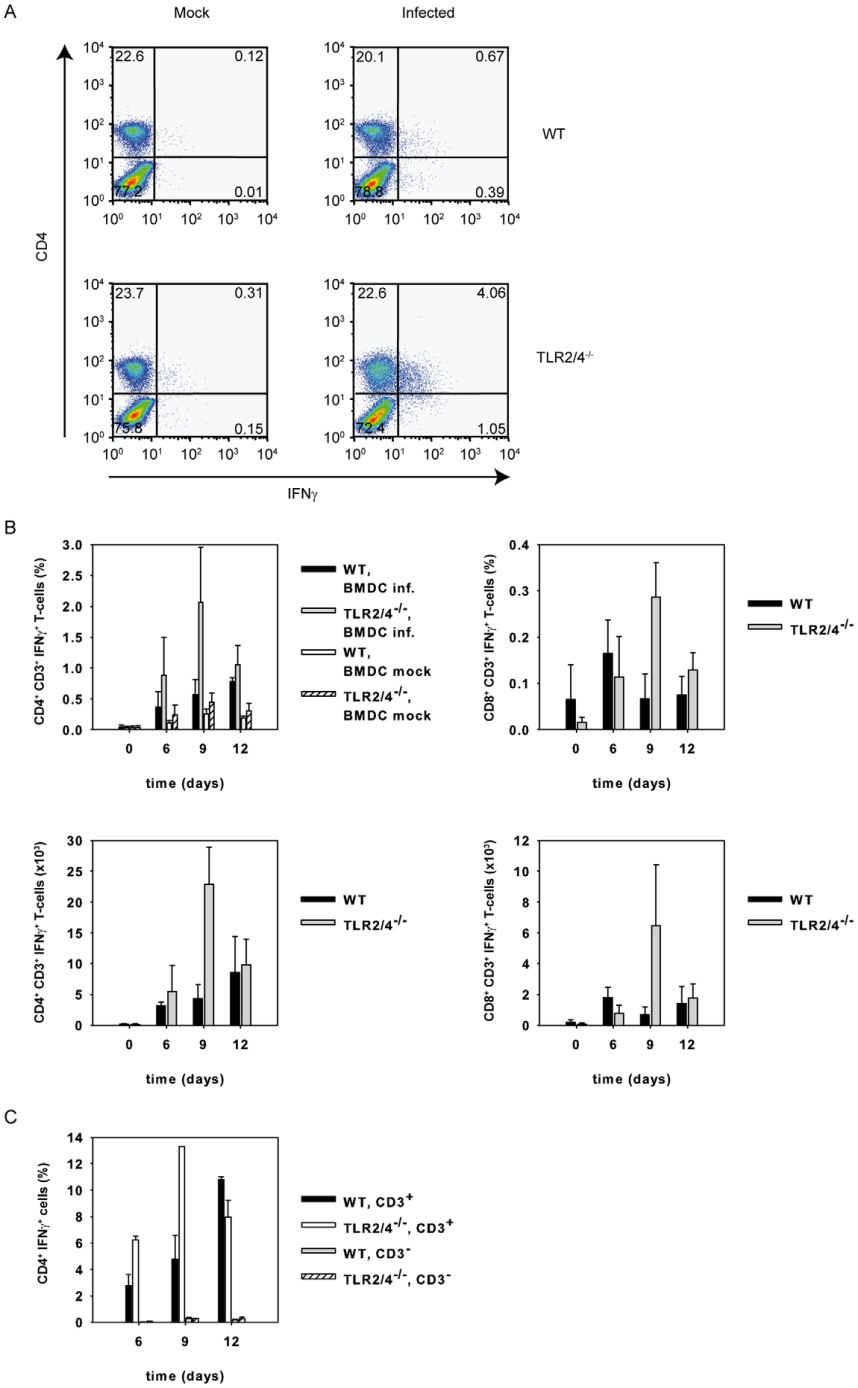
*C. pneumoniae*-specific CD4^+^ T cells are responsible for the enhanced IFNγ-response in TLR2/4 double-deficient mice. (A) Pulmonary cells were prepared from wild type and TLR2/4 double-deficient mice nine days post infection with *C. pneumoniae*. The cells (1×10^5^ cells/well) were then re-stimulated with BMDC (1×10^5^ cells/well) which were or were not infected with *C. pneumoniae* (MOI = 5). After 1 h of culture Brefeldin A was added, the culture continued for another 12 h and cells were stained for CD4, CD8 and intracellular IFNγ. FACS graphs show the IFNγ response of CD4^+^ T-cells. (B) Pulmonary cells from *C. pneumoniae*-infected wild type (n = 3/time point) and TLR2/4 (n = 3/time point) double-deficient mice were prepared at different time points as indicated in the graph. Mock infected animals served as controls (time point 0). Cells were stained with mAbs specific for CD3, CD4, CD8 and IFNγ as described in Materials and Methods and analyzed by flow cytometry. Upper graphs show frequncies, lower graphs absolute numbers of IFNγ^+^CD4^+^ or CD8^+^ T cells. (C) Pulmonary cells were prepared from *C. pneumoniae*-infected wild type (n = 2/time point) of TLR2/4 double-deficient mice (n = 2 6d, n = 1 9d, n = 12d) after 6, 9, and 12 days post infection and the cells were stained for CD4, CD3 and IFNγ. Note that CD4^+^CD3^−^ cells hardly contribute to IFNγ-secretion.

To study whether *C. pneumoniae*-specific T-cells were detectable in the infected lung of TLR2/4 double deficient mice, we re-stimulated T-cells isolated from the lung of infected animals with wild type BMDC *in vitro*, which were either infected or not infected with *C. pneumoniae*. Intracellular staining for IFNγ revealed that the frequency and absolute number of CD4^+^ T-cells producing the cytokine upon re-stimulation with *C. pneumoniae* were higher in TLR2/4 double-deficient when compared to wild type mice especially day 9 post infection (Fig. 3A, B). Compared to CD4^+^IFNγ^+^ T-cells, the frequency and absolute number of IFNγ-secreting CD8^+^ T-cells were considerably lower in both strains of mice (Fig. 3B). However, on day 9 these cells were also enhanced in frequency in the infected TLR2/4 double-deficient mice. Furthermore, IFNγ-secretion by CD4^+^ but CD3^−^ non T-cells was barely detectable (Fig. 3C). These results suggested that antigen-specific TH1 T-cell responses were efficiently generated in the absence of TLR2 and 4.

### Enhanced *Chlamydia*-specific IFNγ-responses in the absence of TLR2/4 is not confined to the lung

**Figure 4:**
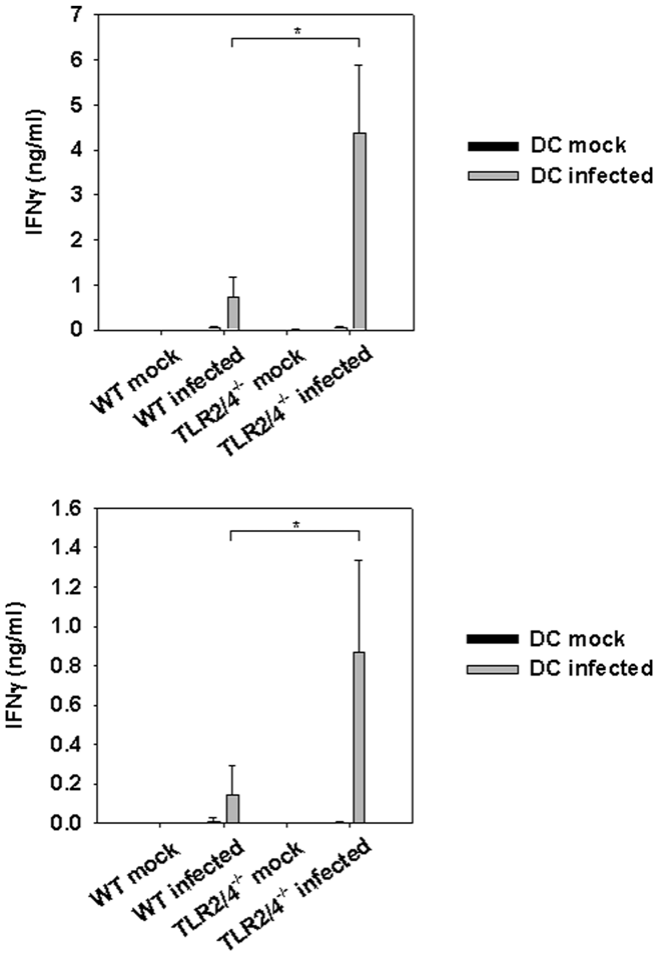
Enhanced extra-pulmonary IFNγ responses in *C. pneumoniae*-infected TLR2/4 double-deficient mice. Lung (upper graph) and spleen cells (lower graph) were prepared nine days post infection with *C. pneumoniae*. Subsequently cells (1×10^5^ cells/well) were re-stimulated with BMDC (1×10^5^ cells/well) which were or were not infected with *C. pneumoniae* (MOI = 5) for three days. The secretion of IFNγ was determined by analyzing the culture supernatant with a commercially available ELISA-system. The graph represents the data of three independent experiments. Error bars represent SD of individual mice (n = 3 mock wild type and mock TLR2/4 double deficient mice, n = 5 infected wild type mice, n = 4 infected TLR2/4 double-deficient mice). *p = 0.016, Mann-Whitney Rank sum test.

Next, we assessed the local and systemic IFNγ-responses from lung and spleen cells of infected wild type or TLR2/4 double-deficient mice upon re-stimulation *in vitro* with *C. pneumoniae*-pulsed wild type BMDC. The results revealed that antigen-specific responses from lung and spleen cells isolated from infected TLR2/4 double-deficient mice secreted considerably more IFNγ than wild type cells after 3 days in culture (Fig. 4). These findings also suggested that increased responses by TLR2/4 double-deficient cells were not confined to the organ infected primarily.

### TLR2/4 double-deficient BMDC are capable of stimulating antigen-specific IFNγ-responses

**Figure 5:**
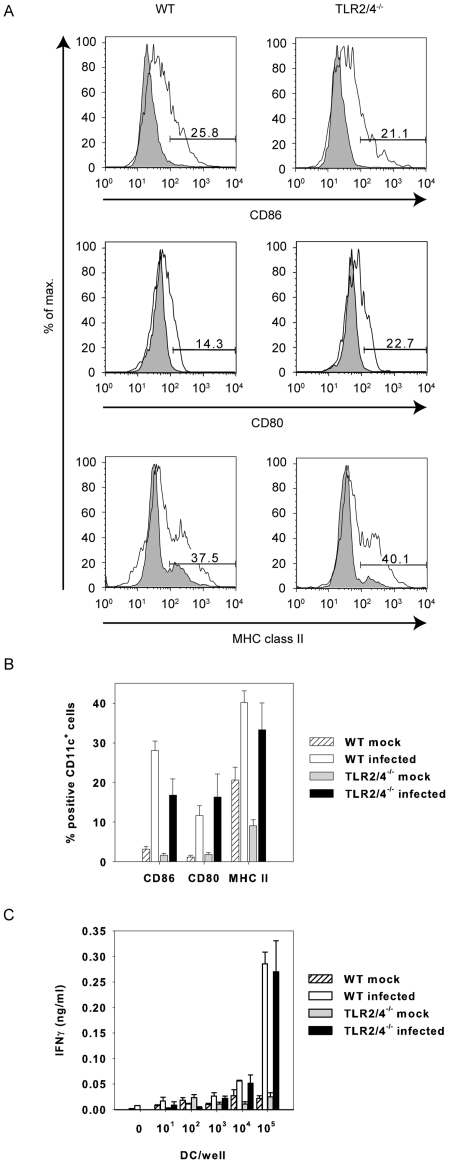
Antigen-presentation is not impaired in TLR2/4 double-deficient BMDC. In (A) and (B) lung cells were prepared three days post infection with *C. pneumoniae*. The cells were stained with antibodies specific for CD11c, CD86, CD80 or MHC class II. All events are gated on CD11c. Error bars represent SD of three individual mice. The experiment was repeated once with similar results. (C) Pulmonary cells were prepared from wild type mice infected nine days earlier with *C. pneumoniae*. The cells (1×10^5^ cells/well) were re-stimulated with titrated amounts of BMDC from wild type or TLR2/4 double-deficient mice as indicated which were or were not infected with *C. pneumoniae* (MOI = 5). The IFNγ-content of the culture supernatant was analyzed by ELISA after three days of culture. Error bars represent SD of three replicate cultures. The experiment was repeated twice with similar results.

We then explored the capacity of TLR2/4 double-deficient BMDC to trigger an IFNγ response by *C. pneumoniae*-specific lung cells. We expected a weaker ability since activation of TLR2-deficient BMDC upon infection with the bacterium *in vitro* was impaired as analyzed by IL-12p40 and TNF secretion and induction of NF-κB [9]. *In vivo*, however, the infection induced the expression of the co-stimulatory molecules CD86 and CD80 as well as MHC class II by CD11c^+^ cells in wild type and TLR2/4 double-deficient mice with comparable efficiency (Fig. 5A, B). Based on the data presented in Fig. 3 we conclude that TLR2/4 double-deficient antigen-presenting cells triggered primary CD4^+^ T cells at least as efficiently as wild type cells. In addition, *C. pneumoniae*-infected TLR2/4 double-deficient BMDC re-stimulated lung cells, which were isolated from *C. pneumoniae*-infected wild type mice, as efficiently as wild type BMDC to secrete IFNγ (Fig. 5C). We therefore conclude that a deficiency of TLR2 and TLR4 by BMDCs did not explain the stronger IFNγ-response of antigen-specific T-cells.

### Lungs of TLR2/4 double-deficient mice contain fewer CD4^+^CD25^+^Foxp3^+^ regulatory T-cells upon infection with *C. pneumoniae*

**Figure 6:**
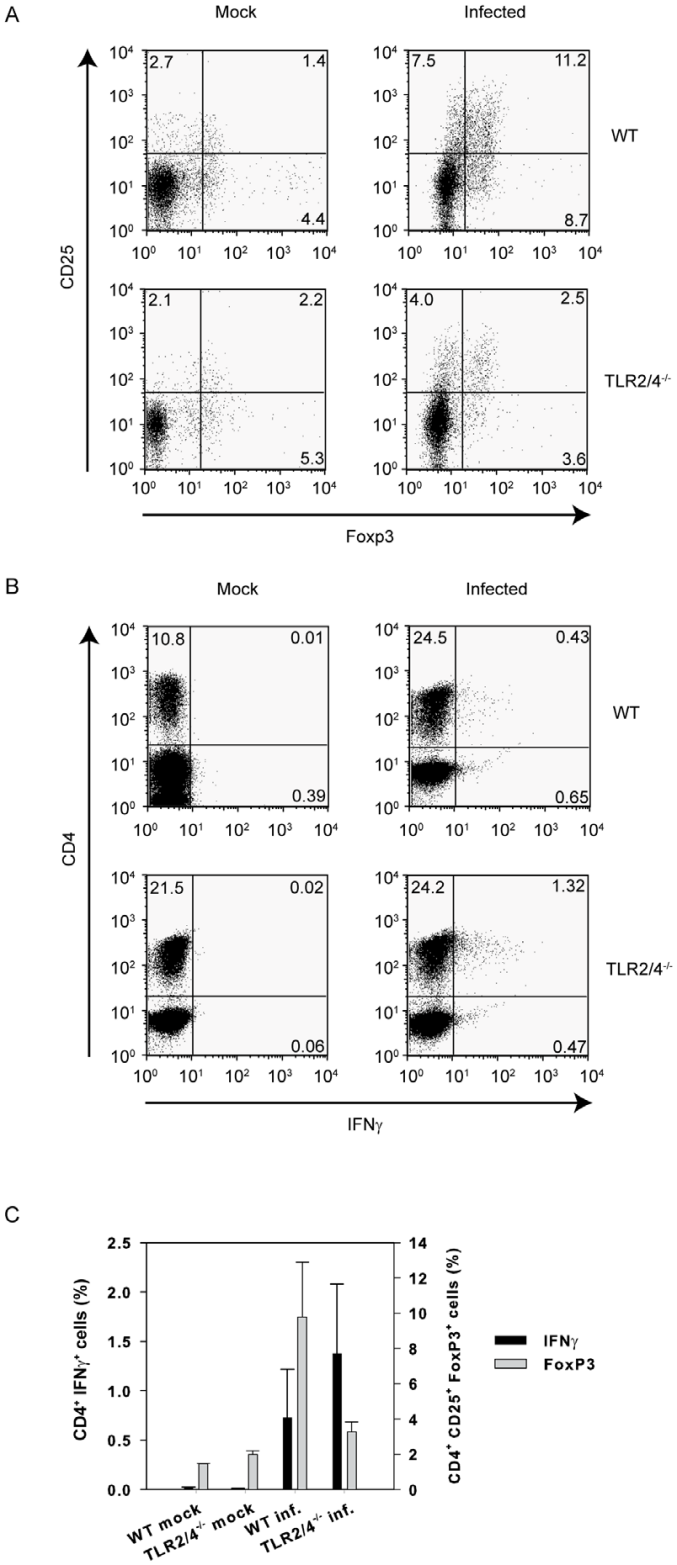
Inverse relation *in vivo* between the percentage of CD4^+^CD25^+^Foxp3^+^ T-cells and the percentage of CD4^+^IFNγ^+^ T-cells. Pulmonary cells were prepared from wild type and TLR2/4 double-deficient mice nine days post infection with *C. pneumoniae*. In (A) the expression of CD25 on the cell membrane and intracellular Foxp3 was analyzed. All events were gated on CD4. (B) shows the expression of intracellular IFNγ by CD4^+^ T-cells *ex vivo* without re-stimulation. (C) demonstrates the relationship between CD4^+^Foxp3^+^ and CD4^+^IFNγ^+^ T-cells. Error bars represent SD of three individual mice. The experiment was repeated twice with similar results.

Several reports indicated that TLR2 influences the expansion and function of regulatory T-cells and express TLR2 on their cell surface upon activation with anti CD3 [14]. Additionally, co-stimulation with the TLR2-ligand Pam3Cys has been shown to induce their proliferation which is accompanied by a transient loss of their suppressive activity [14], [15]. *In vivo*, TLR2 was also shown to be crucial for the expansion of regulatory T-cells in mice infected with *Schistosoma mansoni* [16]. Therefore, we analyzed the frequency of CD4^+^CD25^+^Foxp3^+^ T-cells in lungs of *C. pneumoniae*-infected wild type and TLR2/4 double-deficient mice. In mock-treated mice the percentage of CD4^+^CD25^+^Foxp3^+^ T-cells was not different in both strains (Fig. 6A, left panels). However, upon infection the frequency of these cells was almost 3-fold higher in wild type compared to TLR2/4 double-deficient mice (Fig. 6A right panels, 6C). Conversely, the percentage of IFNγ-producing CD4^+^ T-cells analyzed *ex vivo* (i.e. the cells were not re-stimulated) was considerably increased in the latter mice (Fig. 6B). Thus, there is an inverse relation between the frequencies of CD4^+^CD25^+^Foxp3^+^ regulatory T-cells and CD4^+^IFNγ^+^ effector T-cells (Fig. 6C).

### IFNγ fails to induce iNOS in *C. pneumoniae*-infected TLR2/4 double-deficient BMDMs

**Figure 7:**
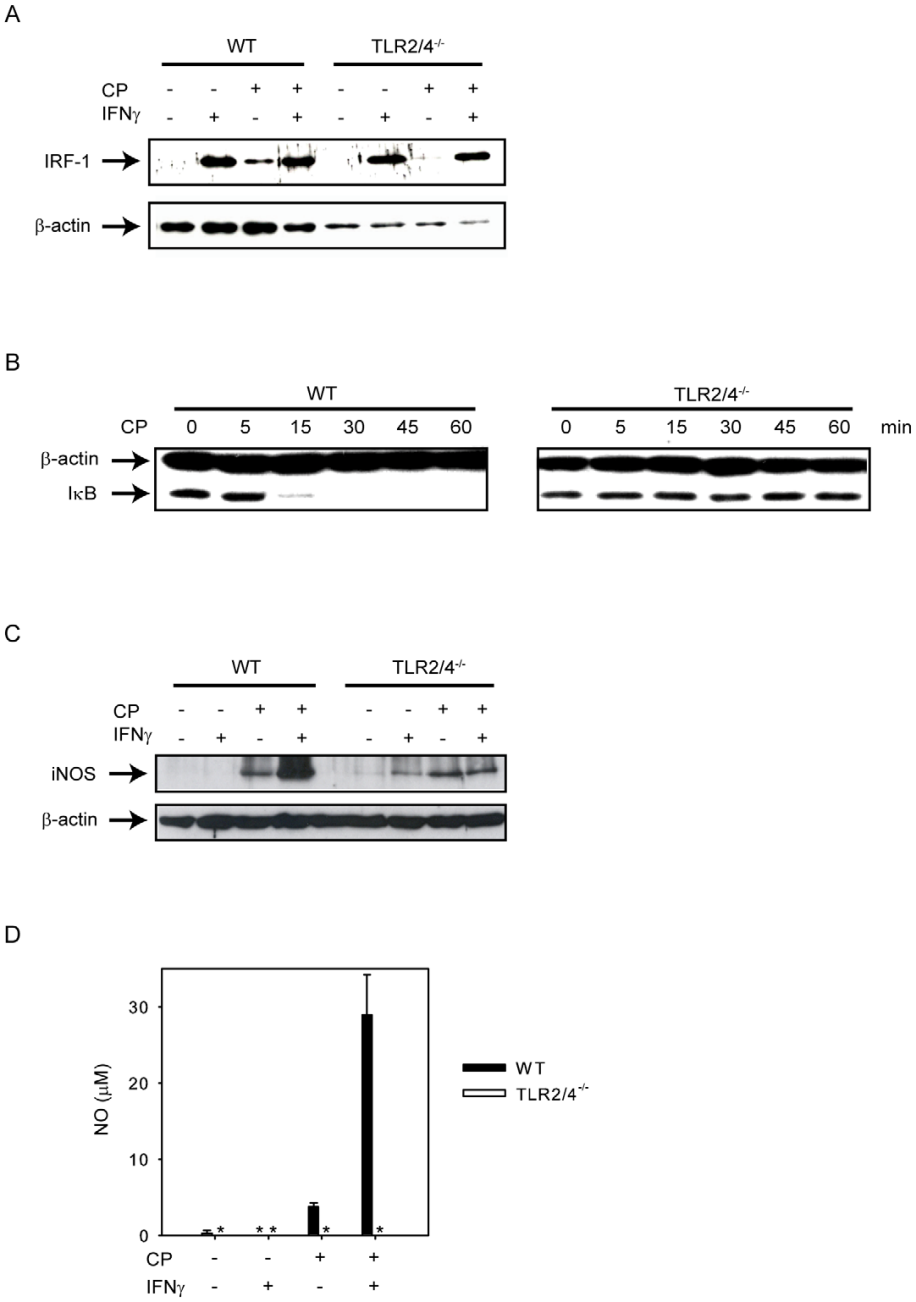
IFNγ is severely impaired to increase iNOS expression and fails to release NO in *C. pneumoniae*-infected TLR2/4 double-deficient BMDMs. (A) Wild type or TLR2/4 double-deficient BMDMs (7.5×10^5^ cells/well) were left untreated, or stimulated with IFNγ (10 ng/ml) for 24 h, or were infected with *C. pneumoniae* (MOI = 10, for 48 h), or were infected with *C. pneumoniae* for 48 h and treated with IFNγ 24 h post infection. IRF-1 was detected by Western blot, detection of β-actin was used as loading control. (B) Wild type or TLR2/4 double-deficient BMDMs (7.5×10^5^ cells/well) were or were not infected with *C. pneumoniae* (MOI = 10) for the time periods indicated in the graph. IκB was detected by Western blot, detection of β-actin was used as loading control. (C) Wild type or TLR2/4 double-deficient BMDMs were treated as described in (A). iNOS was determined by Western blot, detection of β-actin was used as loading control. (D) Wild type or TLR2/4 double-deficient BMDMs (7.5×10^5^ cells/well) were left untreated, or stimulated with IFNγ (10 ng/ml) for 48 h, or were infected with *C. pneumoniae* (MOI = 10, for 72 h), or were infected with *C. pneumoniae* for 72 h and treated with IFNγ 48 h post infection. Subsequently, NO-levels were determined in the culture supernatant. Error bars represent SD of four or in the case of untreated samples of two individual cultures. *not detectable. The experiment was repeated once with similar results.

Normally, CD4^+^IFNγ^+^ effector T-cells play a protective role during *C. pneumoniae* infection [8]. As demonstrated in Fig. 1, TLR2/4 double deficient mice lost considerably more weight at day 12 post infection than wild type mice despite increased IFNγ levels. We also demonstrated earlier that these mice displayed a higher lethality [10]. Therefore, we were interested in the effects of IFNγ on anti-chlamydial defense and investigated the influence of IFNγ on iNOS production in TLR2/4 double-deficient cells. This enzyme participates in the control of an infection with *C. pneumoniae in vivo* as revealed by the analysis of iNOS-deficient mice [7]. We also examined previously the role of MyD88, the adapter molecule used by TLR2 and TLR4, in the induction of iNOS and showed that the level of this enzyme was reduced in infected MyD88-deficient mice [17]. MyD88-deficient BMDM failed to release nitric oxide (NO) upon stimulation with *C. pneumoniae* and IFNγ since two important transcription factors, NF-κB and AP-1 which participate in the transcriptional regulation of the *nos2* gene, were not induced, while induction of IRF-1 and phosphorylation of STAT-1 were normal [17]. As we show here, upon stimulation with IFNγ IRF-1 induction is not affected in TLR2/4 double-deficient macrophages (Fig. 7A). Moreover, prior infection of the macrophages with *C. pneumoniae* did not alter the ability of IFNγ to induce IRF-1 in BMDM of both genotypes (Fig. 7A). As expected, infection of BMDM with *C. pneumoniae* degraded IκB in wild type but not in TLR2/4 double-deficient cells (Fig. 7B). Since NF-κB is crucial for the induction of iNOS [17], it was not unexpected that the ability of IFNγ to induce iNOS-expression was severely impaired in *C. pneumoniae*-infected TLR2/4 double-deficient BMDM (Fig. 7C). Moreover, NO-secretion was completely abolished (Fig. 7D). Taken together, the failure of IFNγ to induce iNOS presumably contributes to lethality observed in TLR2/4 double-deficient mice.

## Discussion

Using TLR2/4 double-deficient mice we show that TLR2 and TLR4 regulate IFNγ-secretion *in vivo* during pneumonia caused by *C. pneumoniae*. Our findings demonstrate that CD4^+^ T-cells were responsible for the enhanced IFNγ-production, since CD4^+^CD3^+^IFNγ^+^ cells were more frequent in lungs of infected TLR2/4 double-deficient mice. IFNγ-producing CD4^+^ T-cells were antigen-specific, since release of IFNγ *in vitro* required re-stimulation of the cells with *C. pneumoniae*-infected BMDC. Enhanced IFNγ-secretion was not confined to CD4^+^ T cells isolated from the infected lungs but was also observed in lymphocytes prepared from the spleen. Antigen-presenting cells appeared not to be involved in enhanced IFNγ-secretion by CD4^+^ T-cells since BMDC from TLR2/4 double-deficient mice re-stimulated wild type cells as efficiently as wild type BMDC. However, upon infection TLR2/4 double-deficient mice displayed a lower frequency of CD4^+^CD25^+^Foxp3^+^ regulatory T-cells *in vivo* and the frequency of regulatory T-cells was inversely related to the frequency of CD4^+^IFNγ^+^ T-cells. Despite the fact that IFNγ was produced in increased amounts by TLR2/4 double-deficient CD4^+^ T-cells, the cytokine failed to induce NO by BMDM lacking TLR2 and TLR4.

Previous reports indicated that the TLR key adapter molecule MyD88 was required for a TH1 CD4^+^ T-cell response upon stimulation of the cells with ovalbumin in complete Freund's adjuvant or by endotoxin plus allogeneic cells [11], [12]. Our results show, however, that in the absence of the relevant TLRs, i.e. TLR2 and TLR4, CD4^+^ T-cells can be sensitized to release IFNγ upon infection with *C. pneumoniae*. In agreement with the data presented here, infection of MyD88-deficient mice with *Mycobacterium bovis* BCG also triggered CD4^+^ T-cells to produce IFNγ [13]. While the reasons for these discrepancies are unclear, it is likely that MyD88-independent recognition systems including NOD-like receptors also recognize bacteria-derived ligands which are presumably not present in complete Freund's adjuvant or in endotoxin preparations. Based on our previous report, *C. pneumoniae*-induced secretion of the cytokines TNF and IFNγ and the chemokines KC and MCP-1 depended on MyD88 three days post infection. However, six days post infection these cyto- and chemokines were secreted MyD88-independently [2]. Thus, we speculate that the complex composition of pathogen-associated molecular patterns of bacteria allows the MyD88-independent induction of a TH1-response.

TLR2/4 double-deficient BMDC re-stimulated wild type cells with the same efficiency as wild type BMDC to produce IFNγ. Although TLR2-deficient BMDC were impaired to secrete TNF and IL-12p40 upon infection with *C. pneumoniae in vitro* [9], both factors were presumably not required for T-cell stimulation. However, TLR4-deficient BMDC failed to increase the expression of the co-stimulatory cell surface molecule CD40 upon stimulation with endotoxin and were unable to stimulate allogeneic T-cells, while the endotoxin-induced up-regulation of CD40, CD80 and CD86 was independent from MyD88 [18]. Since the infection of TLR2/4 double-deficient mice with *C. pneumoniae* generated even more CD4^+^IFNγ^+^ T-cells, we conclude that *in vivo* antigen-presenting cells are not impaired in their ability to activate antigen-specific T-cells in the absence of TLR2 and TLR4.

Enhanced IFNγ-responses by *C. pneumoniae*-specific CD4^+^ T-cells in TLR2/4 double-deficient mice are best explained by an impaired infection-associated expansion of CD4^+^CD25^+^Foxp3^+^ regulatory T-cells. In contrast to MyD88-deficient mice, which appear to contain reduced numbers of regulatory T-cells [15], the frequency of these cells in mock-treated TLR2/4 double-deficient mice was similar to the one in wild type mice. Our results are in agreement with reports, which show that expansion of Tregs can be triggered by a co-stimulation of T-cell receptors and TLR2 [14], [15]. As we have published earlier, chlamydial heat shock protein 60 (HSP60, GroEL) triggers cells via TLR2 and TLR4 [19], [20], [21] and may (together with *C. pneumoniae*-derived antigens) be responsible for the expansion of CD4^+^CD25^+^Foxp3^+^ Tregs *in vivo*.

Our results also imply that the adaptive TH1 immune response is unable to prevent the lethal outcome of pulmonary infection caused by *C. pneumoniae*, if TLR2 and TLR4 are absent. In association, previous studies have shown the importance of IFNγ-producing CD4^+^ T-cells in increasing resistance of RAG-1/IFNγ-double-deficient mice against an infection with *C. pneumoniae* [8]. Similar observations were obtained in a model where MyD88-deficient mice were infected with *M. tuberculosis*. Vaccination of MyD88-deficient mice with *M. bovis* BCG induced IFNγ-producing CD4^+^ T-cells *in vivo*, but the TH1 cells only partially prevented lethal pneumonia upon challenge with *M. tuberculosis* [13]. Upon challenge with *C. pneumoniae* MyD88-deficient mice succumbed like TLR2/4-deficient mice to progressive pneumonia [10], although pulmonary IFNγ-levels were increased like in wild type mice six days post infection [2]. Furthermore, the ability of IFNγ to induce iNOS was impaired in MyD88-deficient macrophages [17]. Full induction of iNOS required the transcription factors AP-1, NF-κB, IRF-1 and STAT-1. The former two were not activated in MyD88-deficient bone marrow-derived macrophages (BMDM) upon infection with *C. pneumoniae in vitro* leading to a complete failure to induce iNOS. *In vivo*, iNOS-levels were considerably reduced in MyD88-deficient mice post infection with *C. pneumoniae*. Identical to these published findings we show here, that in *C. pneumoniae*-infected TLR2/4 double-deficient BMDM IFNγ only weakly up-regulated the expression of iNOS and failed to induce the secretion of NO, since the infection of these cells with *C. pneumoniae* did not induce NF-κB. INOS is crucial to control the replication of *C. pneumoniae in vivo*, since the chlamydial burden is considerably increased in iNOS-deficient mice [7]. Thus, although *C. pneumoniae*-specific CD4^+^ T-cells produce enhanced amounts of IFNγ in TLR2/4-deficient mice, the cytokine cannot completely exert its protective effects in TLR2/4 double-deficient mice, since it is impaired to induce iNOS.

In summary, expansion of CD4^+^CD25^+^Foxp3^+^ T-cells is impaired in *C. pneumoniae*-infected TLR2/4 double-deficient mice. This is accompanied by an increased frequency of CD4^+^IFNγ^+^ effector T-cells which cannot prevent lethal pneumonia.

## Materials and Methods

### Ethic statement

All animal experiments were reviewed and approved by the local authorities (Regierung von Oberbayern, file number 211-2531-59/06).

### Strains of mice

C3H/HeN mice were purchased from Harlan Winkelmann GmbH (Borchen, Germany). Breeding pairs of C57BL/6 TLR2^−/−^ mice came from Tularik (South San Francisco, CA). They were backcrossed six times to TLR4^d/d^ C3H/HeJ mice to generate TLR2/4 double-deficient mice. The genotype of the mice was verified phenotypically as described in [21]. Alternatively, TLR2^−/−^ mice were crossed with TLR4^−/−^ mice and the resulting TLR2/4 double-deficient mice were further backcrossed nine times to C3H/HeN mice. The genotype of these mice was analyzed by PCR (Fig. S1). All mice were bred in our own animal facility under specific pathogen-free conditions.

### Reagents

The peroxidase-conjugated AffiniPure 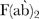 fragment Donkey anti-Rabbit IgG (H+L) and peroxidase-conjugated AffiniPure 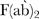 fragment Goat anti-Mouse IgG+IgM (H+L) were purchased from Dianova (Germany), and the monoclonal antibody to β-actin was provided by Sigma-Aldrich (Germany). The monoclonal antibodies specific for IRF-1 (M-20): sc-640, iNOS and IκB (clone E130) were provided by Santa Cruz Biotechnology, Inc. (CA, USA), Upstate (Millipore, Germany) and Epitomics (USA), respectively. Murine IFNγ (315-05) was purchased from Peprotech Inc. (New Jersey, USA).

### Infection protocol

Anesthesized mice were infected intranasally with *Chlamydia pneumoniae* (CM-1, ATCC VR-1360, 2.5×10^6^ IFUs). Infected mice were inspected every day and body weight was determined on day 6 and 9 and 12 post infection.

### Generation of BMDC and BMDM

BMDC were generated according to Inaba et al with slight modification [22]. Mice were sacrificed and tibiae and femora were removed, cleaned and flushed with cell culture medium. They were plated on bacterial petri-dishes overnight in culture medium (RPMI 1640, 10% heat-inactivated FCS, 100 µg/ml penicillin, 100 µg/ml streptomycin, 5×10^−5^ M 2-ME) to remove adherent cells. Non-adherent cells were plated at a density of 5×10^6^ cells/dish, and cultivated for another five days in complete medium in the presence of GM-CSF (10% v/v). The cells were counted, plated in medium devoid of FCS and antibiotics and exposed to *C. pneumoniae* at a multiplicity of infection (MOI) of 5. After 30 hours the BMDC were counted and used for re-stimulation assays.

BMDM were generated according to Rutschman et al. [23]. Briefly, femora and tibiae of mice were rinsed with cell culture medium applied through a 26-gauge syringe. Bone marrow cells were cultured in petri dishes at a density of 5×10^6^ cells/dish in the presence of L cell-conditioned medium as a source of M-CSF-1 (10% v/v). The medium used was very low endotoxin DMEM (PAA Laboratories GmbH, Austria) supplemented with 10% FCS (Biochrom AG, Germany), 2-ME (50 µM; Life Technologies, Germany) and the antibiotics Vancomycin and Gentamicin, both provided by Sigma-Aldrich (Germany). Cells were washed vigorously and only adherent macrophages were used 6–7 days after plating. FACS analysis showed that these BMDM were F4/80^+^ and CD11b^+^ as described previously (data not shown) [10].

### Isolation of lung and spleen cells

Mice were sacrificed with CO_2_ on day 6, 9 or 12 post infection with *C. pneumoniae*. Lungs were flushed with 10 ml PBS and digested after manual mincing. The lungs were digested with collagenase VIII (400 U/100 µl 10 min at room temperature, 400 U/2 ml RPMI 0% FCS, 30 min at 37°C), washed and separated through a cell strainer. Spleens were removed and a single cell suspension was prepared. Erythrocytes were lysed with ammonium chloride (0.15 M, 5 min, RT) and washed subsequently with PBS containing 3% FCS. Co-cultures for intracellular staining were performed with 4×10^6^ lung cells and 1×10^6^ BMDC for 13 h. Brefeldin A (Golgi-Plug, BD Biosciences, San Jose, CA, USA) was added for the last 12 h of culture. For long-term cultures, 1×10^5^ pulmonary or spleen cells and 1×10^5^ BMDC were co-cultured in 96-well plates.

### Determination of IFNγ levels

IFNγ levels were determined using a commercially available ELISA system (DuoKit BD Biosciences). The assay was performed according to manufacturer's manual. Data were analyzed using SigmaPlot 10.0 (Systat Software, Inc.).

### Flow cytometry

Cells were counted and concentrated to 2×10^6^ cells per ml. Cells were washed with PBS (3% FCS) and pre-incubated with EMA (2 µg/ml, 20 min 4°C, Molecular Probes, OR, USA) to exclude dead cells and anti-CD16/32 (10 µg/ml, 10 min, 4°C, Caltag Laboratories, Invitrogen, Carlsbad, CA, USA) to block Fc-receptors. Cells were stained with different combinations of APC- or PE-labelled mAb specific for CD3, APC-labelled mAb directed against CD8 (2 µg/ml, Caltag Laboratories) and PE-labelled mAb directed against CD4 (2 µg/ml, Caltag Laboratories) for 30 min on ice. The cells were washed again and then fixed and permeabilized with Perm/Wash (BD Biosciences, San Jose, CA, USA) for 20 min on ice. After another wash step intracellular staining was performed with FITC-labelled mAb directed against IFNγ (1 µg/ml, BD) for 30 min on ice. Cells were washed with Perm/Wash (BD) twice and fixed again with 2% paraformaldehyde.

To detect regulatory CD4^+^ T-cells, cells were also stained with a mAb specific for CD25 (5 µg/ml, 30 min, 4°C, BD) and with a mAb specific for Foxp3 (clone FJK-16 s, eBioscience, Frankfurt, Germany) using the Foxp3 Staining Buffer Set from eBioscience (00-5523-00) according to the manufacturer's recommendations.

The induction of co-stimulatory molecules on CD11c^+^ pulmonary cells was analyzed by staining the cells with antibodies specific for CD11c (2 µg/ml, Caltag Laboratories), CD80 (5 µg/ml, BD), CD86 (2 µg/ml, BD) and MHC class II molecules I-A/I-E (5 µg/ml, BD).

Flow cytometry was performed with a Calibur instrument (BD Biosciences, San Jose, CA, USA), the data were analyzed using the FloJo software (Three Star Inc, OR, USA).

### Cell lysis, SDS-PAGE and Western blotting

Cell extracts were prepared for Western blotting using RIPA buffer (20 mM Tris pH 7.5, 150 mM NaCl, 1% NP-40, 0,5% Sodium Deoxycholate, 1 mM EDTA, 0,1% SDS) which was supplemented with Sodium Orto-Vanadat (Sigma-Aldrich, Germany) and a protease inhibitor cocktail (Roche Diagnostics GmbH, Germany). Cell lysates were clarified by sonication and centrifugation before electrophoresis. Proteins were separated by SDS-PAGE (10% acrylamide) in TANK Buffer (25 mM Tris, 0.2 M glycine, 0.1% SDS), using Laemmli buffer (62.5 mM Tris, 50% glycerol, 2% SDS, 2 mM EDTA) for sample loading. After transfer to nitrocellulose membrane by semidry electroblotting for 1.5 h (2 mA/cm^2^) in Transfer Buffer (25 mM Tris, 250 mM Glycine, 20% Methanol, 0,35% SDS), membranes were blocked in TBST (2.4 g/l Tris, 8 g/l NaCl, 0.1% Tween, pH 7.6, containing 5% milk powder, 2 h, room temperature). Thereafter, membranes were incubated with antibodies specific for iNOS (diluted 1∶1000), IRF-1 (diluted 1∶500), IκB (diluted 1∶5000) and β-actin (diluted 1∶20000). All primary antibodies were incubated overnight at 4°C. After three washing steps with TBST the secondary antibody was added (diluted 1∶8000 in TBST containing 5% milk powder, 2 h, room temperature). The blot was washed again three times with TBST and visualized using the Western lightning™- Chemiluminiscence Reagent (Perkin Elmer LAS Inc, MA, USA) as described by the manufacturer.

### Nitrite measurement

Nitrite production was measured from wild type and TLR2/4 double-deficient BMDM supernatants. Briefly, the cells were cultured in 12-well plates in 1 ml of culture medium until confluence. The cells were stimulated with IFNγ or *C. pneumoniae* and the culture supernatants were collected. Nitrite was measured by adding 50 µl of Griess reagent (1% sulfanilamide and 0.1% naphthylethylenediamide in 5% phosphoric acid) to 50 µl samples of culture medium. The optical density at 550 nm (OD_550_) was measured using a microplate reader and the nitrite concentration calculated by comparison with the OD_550_ produced using standard solutions of sodium nitrite in the culture medium.

### Statistics

Comparison of two equally treated groups was analyzed by Mann-Whitney Rank sum test. More than two equally treated groups were tested for significant differences with one way ANOVA, post hoc test Holm-Sidak. Statistical analysis was performed with SigmaStat (SPSS Inc., IL, USA).

## Supporting Information

Figure S1 **Identification of TLR2/4 double-deficient mice.** Genomic tail DNA was prepared from 19 mice bred from a cross of TLR2- or TLR4-deficient mice. Primer sequences used were as follows: TLR2 WT1 5′-CTTCCTGAATTTGTCCAGTACAGG-3′ TLR2 WT2 5′-TCGACCTCGATCAACAGGAGAAGGG-3′ TLR2 KO 5′-GGGCCAGCTCATTCCTCCCACTCAT-3′ TLR4 WT1 5′-GTTTAGAGAATCTGGTGGCTGTGGAGAC-3′ TLR4 WT2 5′-TATATG CGGCCGCTCATCTGC TGTACTTTTTACAGCC-3′ TLR4 KO5′-TGTTGGGTCGTTTGTTCGGATCCGTCG-3′. PCR amplification using primers TLR2 WT1 and TLR2 WT2, or TLR2 WT1 and TLR2 KO, or TLR4 WT1 and TLR4 WT2, or TLR4 WT1 and TLR4 KO detected the wild type TLR2 gene, TLR2-deficiency, the wild type TLR4 gene or TLR4-deficiency, respectively. The different PCRs were run with slight variations (details upon request): 94–95°C180–300 s 94–95°C 30–60 s 59–67°C 30–60 s30 cycles 70–74°C 60–180 s 72°C120 s 4°C.
(TIF)
