# Supplementary figures and images for: Toll-Like Receptors 2 and 4 Regulate the Frequency of IFNγ-Producing CD4+ T-Cells during Pulmonary Infection with Chlamydia pneumoniae
Source: PLoS One. 2011 Nov 9;6(11):e26101. doi: 10.1371/journal.pone.0026101 (PMC3212512; doi:10.1371/journal.pone.0026101)

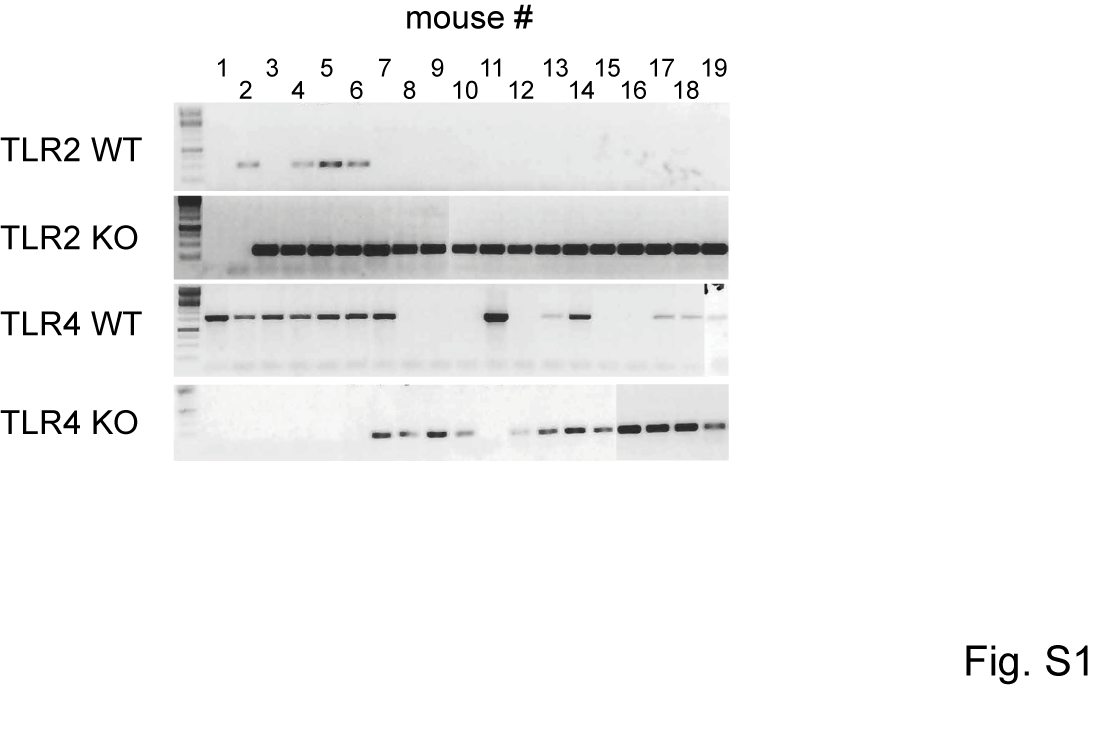

Supplement: Figure S1 — Identification of TLR2/4 double-deficient mice. Genomic tail DNA was prepared from 19 mice bred from a cross of TLR2- or TLR4-deficient mice. Primer sequences used were as follows: TLR2 WT1 5′-CTTCCTGAATTTGTCCAGTACAGG-3′ TLR2 WT2 5′-TCGACCTCGATCAACAGGAGAAGGG-3′ TLR2 KO 5′-GGGCCAGCTCATTCCTCCCACTCAT-3′ TLR4 WT1 5′-GTTTAGAGAATCTGGTGGCTGTGGAGAC-3′ TLR4 WT2 5′-TATATG CGGCCGCTCATCTGC TGTACTTTTTACAGCC-3′ TLR4 KO5′-TGTTGGGTCGTTTGTTCGGATCCGTCG-3′. PCR amplification using primers TLR2 WT1 and TLR2 WT2, or TLR2 WT1 and TLR2 KO, or TLR4 WT1 and TLR4 WT2, or TLR4 WT1 and TLR4 KO detected the wild type TLR2 gene, TLR2-deficiency, the wild type TLR4 gene or TLR4-deficiency, respectively. The different PCRs were run with slight variations (details upon request): 94–95°C180–300 s 94–95°C 30–60 s 59–67°C 30–60 s30 cycles 70–74°C 60–180 s 72°C120 s 4°C. (TIF) [file pone.0026101.s001.tif]
